# Assessing the effects of organizational support, psychological capital, organizational identification on job performance among nurses: a structural equation modeling approach

**DOI:** 10.1186/s12913-023-09705-z

**Published:** 2023-07-27

**Authors:** Huijuan Ma, Xiaoli Zhu, Jinyu Huang, Suofei Zhang, Jing Tan, Yu Luo

**Affiliations:** 1 School of Nursing, Third Military Medical University/Army Medical University, No. 30 Gaotanyan Street, Shapingba District, Chongqing, P.R. China; 2 Institute of Military Preventive Medicine, Third Military Medical University/Army Medical University, Chongqing, P.R. China

**Keywords:** Organizational support, Job performance, Psychological capital, Organizational identification, Mediating effect, Nurse

## Abstract

**Background:**

The job performance of individual employees determines the overall performance of an organization, and organizational support is known as an important resource at the organizational level to enhance job performance. Although nursing scholars have confirmed the crucial role of organizational support in enhancing job performance, there are no studies on whether psychological capital and organizational identification mediate the association between organizational support and job performance. The purpose of this study is to investigate the influence of organizational support, psychological capital, and organizational identification on nurses’ job performance.

**Methods:**

A cross-sectional survey was conducted among 455 nurses from 21 public hospitals in China. Instruments were perceived organizational support scale, task performance scale, contextual performance scale, Nurse Psychological Capital Questionnaire, and Organizational Identification Questionnaire. Survey data were analyzed using SPSS and AMOS, and hypotheses were tested using path model analysis.

**Results:**

Nurses’ perceived organizational support, psychological capital, organizational identification, and task/contextual performance were positively correlated in every two variables. Psychological capital played an important mediating role in perceived organizational support and task/contextual performance, as well as organizational identification. The multi-mediating effect of psychological capital and organizational identification on the relationship between organizational support and task/contextual performance were 0.14 and 0.25, respectively.

**Conclusions:**

There was a positive correlation between organizational support and job performance among nurses. Psychological support, organizational identification and contextual performance played a chain mediation role in the relationship between organizational support on task performance in nurses. Nursing managers should pay more attention to enhancing nurses’ psychological capital and organizational identification through effective interventions to improve nurses’ job performance.

**Supplementary Information:**

The online version contains supplementary material available at 10.1186/s12913-023-09705-z.

## Background

Job performance is a multidimensional concept that is defined as a certain behavior that organizations expect from an individual over a standard period of time [1]. Job performance can be divided into task performance and contextual performance by Borman and Motowidlo [2]. Task performance is behavior that has a direct link with completion of the job, and contextual performance is not directly related to task but can promote task performance [3]. The job performance of individual employees determines the overall performance of an organization [4], and job performance along with the factors affecting job performance has been studied by many researchers over the past decades.

Nurses’ job performance is an important indicator of healthcare organizations’ overall performance, and effective job performance of nurses is reported to have positive effect on patient safety and health outcomes [5]. According to the demands-resources-stressors (JD-R-S) model, resources at the individual level, job level, organizational level, and social level are important factors in job performance [6]. Among them, organizational support is known as an important resource at the organizational level to enhance job performance [4]. Organizational support is a concept derived from the theory of organizational support theory and social exchange theory [7], which can contribute to the attachment of employees to the organization physically and emotionally, and which can affect the employees’ job performance and the success of the organization [8, 9]. In nursing settings, organizational support is also significantly related to nurses’ task performance as shown in the study conducted in Saudi Arabia [10]. However, more evidence is needed to fully illuminate the relationship between organizational support and nurses’ job performance.

In addition to organizational support, positive psychological capital is also reported to have positive effect on nurses’ performance [11]. With emotional and physical support from the organization, employees believe that their efforts will be rewarded and that they will be given the opportunity to succeed and grow in the organization [12]. In these circumstances, psychological capital can be increased by developing organizational support [4]. As a positive psychological state, psychological capital can motivate individuals to accomplish tasks and improve job performance [13]. Consistent with the above theory, psychological capital is known to play a mediating role in the relationship between organizational support and nurses’ job performance [14, 15].

Strong organizational identification is also reported to have a positive effect on nurses’ performance [16]. Meanwhile, a positive relationship between organizational support and organizational identification is demonstrated in an organizational context [17, 18]. Past research has also shown that organizational support and organizational identification, which are psychologically connected and bonded, play a crucial role in the growth and development of organization [7]. Organizational identification is based on social identity theory, which is the extent to which employees identify themselves with the organization [19, 20]. With a higher level of organizational identification, employees value their organizations and have the motivation to achieve bigger efforts towards the goals of organization [21]. The degree of organizational identification has a positive association with the level of job performance [19]. Although the influence of organizational support on work performance through organizational identification among company employees has been demonstrated in past research [22], more evidence is needed in the nursing context.

From the above literature, it can be seen that both psychological capital and organizational identification are important mediating variables between organizational support and job performance. Interestingly, psychological capital as a core construct has been shown to have positive outcomes on organizational identification [23]. Therefore, it is hypothesized that psychological capital and organizational identification have a chain mediating effect on the relationship between organizational support and job performance.

Because strong organizational support can promote better work outcomes and job performance in nurses [24], the mechanism of the above relationship is given attention by nursing scholars. Gillet et al. tested a model linking procedural justice, supervisor autonomy support, need satisfaction, organizational support, work satisfaction, organizational identification, and job performance [25], and Hafidhah and Martono explored the effect of perceived organizational support, job stress, and organizational culture on nurses’ job performance [26]. In addition, AKKOÇ and YILMAZ investigated the mediating role of trust in the relationship between organizational support and job performance in nurses [24]. However, there is no evidence on the effect of organizational support on nurses’ job performance when psychological capital and organizational identification are included. This study attempts to explore how organizational support affects nurses’ job performance from the perspective of psychological capital and organizational identification.

### Hypotheses

**Fig. 1:**
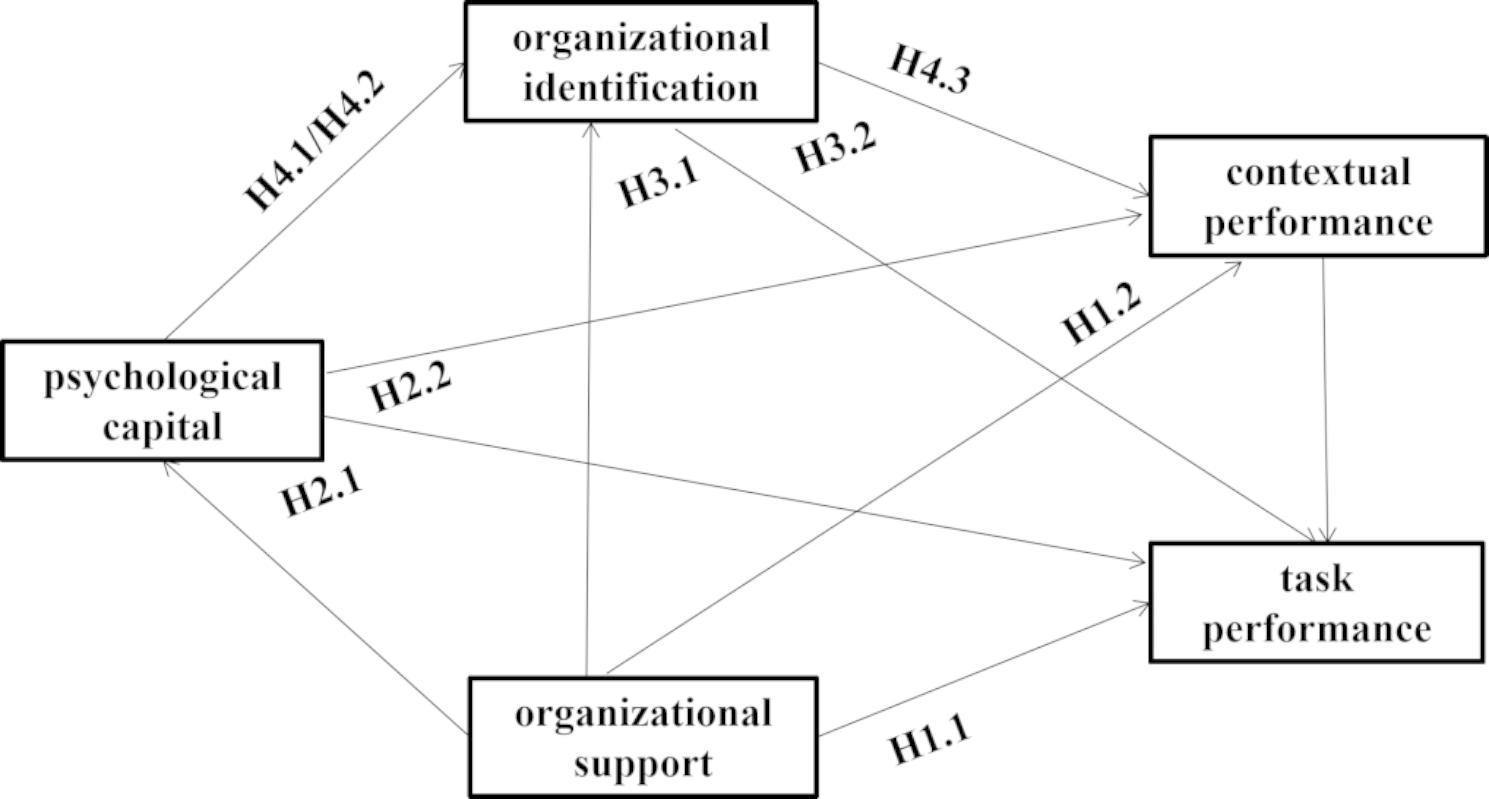
Hypothesized model

Hypothesized model of the chain mediating effect of psychological capital and organizational identification on the relationship between organizational support and nurses’ job performance was proposed, as shown in Fig. 1. This hypothesized model consisted of nine hypotheses.

#### Hypothesis 1.1

*Organizational support is positively correlated with nurses’ task performance.*

#### Hypothesis 1.2

*Organizational support is positively correlated with nurses’ contextual performance.*

#### Hypothesis 2.1

*Psychological capital mediates the positive link between organizational support and task performance.*

#### Hypothesis 2.2

*Psychological capital mediates the positive link between organizational support and contextual performance.*

#### Hypothesis 3.1

*Organizational identification mediates the positive link between organizational support and task performance.*

#### Hypothesis 3.2

*Organizational identification mediates the positive link between organizational support and contextual performance.*

#### Hypothesis 4.1

*Psychological capital and organizational identification mediates the relationship between organizational support and task performance sequentially.*

#### Hypothesis 4.2

*Psychological capital and organizational identification mediates the relationship between organizational support and contextual performance sequentially.*

#### Hypothesis 4.3

*Psychological capital, organizational identification, and contextual performance mediates the relationship between organizational support and task performance sequentially.*

## Methods

### Samples and procedure

A cross-sectional questionnaire survey was conducted from September to December 2020 among 21 public hospitals in northern, western, southern, and eastern China, and convenience sample method was adopted. Prior to the survey, the research team contacted nursing managers of public hospitals from different regions in China, and nursing managers helped in release recruitment information. The eligibility criteria were as follows: (1) registered nurses working in the public hospitals, (2) more than 1 year of nursing experience, (3) willing to participate in the survey. Considering a minimum sample size of 200 for structural equation model [27] and rate of invalid questionnaires being 20%, at least 240 questionnaires should be collected. The research team introduced the aim and design to nurses who wanted to participate, and questionnaires were distributed through online platforms after obtaining their approval. If any item was blank when participants submitted the questionnaire, they would be reminded to fill the blanks. A total of 476 nurses completed the questionnaire, and 21 invalid questionnaires were deleted because the answers to various items in the questionnaire had obvious regularity and the questionnaires of the same hospital were clearly similar. A sample of 455 nurses was obtained with a mean age being 36.89 ± 6.058 years and mean nursing work experience being 14.48 ± 7.311. Of the participants, 446 (98.02%) were female, 395 (86.81%) were married, 385 (84.62%) had bachelor degree, 229 (50.33%) were nurse managers, see Table 1.

**Table 1:** Participant characteristics

Characteristics	Categories	n (%)
Gender	Female	446 (98.02)
	Male	9 (1.98)
Marital status	Unmarried	45 (9.89)
	Married	395 (86.81)
	Divorced	15 (3.30)
Professional Title	Nurse	16 (3.52)
	Senior nurse	163 (35.82)
	Nurse in charge	251 (55.16)
	Associate director	21 (4.62)
	Director of nurses	4 (0.88)
Age (years)	≤ 29	53 (11.65)
	30 ~ 39	275 (60.44)
	≥ 40	127 (27.91)
Education status	College degree or lower	47 (10.33)
	Bachelors	385 (84.62)
	Masters or higher	23 (5.05)
Position	Nurse	226 (49.67)
	Nurse manager	229 (50.33)
Clinical career (years)	≤ 5	59 (12.97)
	6 ~ 10	71 (15.60)
	11 ~ 15	162 (35.60)
	16 ~ 20	76 (16.70)
	≥ 21	87 (19.12)
Department	Medicine	139 (30.55)
	Surgical	157 (34.51)
	Others	159 (34.95)

### Instruments

Instruments used in this study were perceived organizational support scale, task performance scale, contextual performance scale, Nurse Psychological Capital Questionnaire, and Organizational Identification Questionnaire (Appendix), and all instruments were the Chinese version.

#### Organizational support

The Chinese perceived organizational support scale [28] was developed on the basis of organizational support theory proposed by Eisenbergerd [29], and perceived organizational support scale for nurses was adjusted by Zuo on the basis of the above Chinese version scale [30]. This scale consists of 13 items in 2 dimensions: emotional support and instrumental support. Each item is rated on a 5-point Likert scale, with higher scores indicating higher levels of perceived organizational support. The reliability of the instrument in this study is 0.983.

#### Job performance

To measure job performance, task performance scale [31] and contextual performance scale [32] were used. The task performance scale is a four-item scale developed by Chinese scholar Fan and Zheng [31], and the contextual performance scale is a 15-item scale developed by Motowidlo and VanScotter [32], which is constituted by interpersonal facilitation (7 items) and job dedication (8 items). Each item is rated on a 5-point Likert scale, with higher scores indicating higher levels of task/contextual performance. The reliability of the task performance scale and contextual performance scale in this study are 0.870 and 0.956, respectively.

#### Psychological capital

To measure psychological capital, the Nurse Psychological Capital Questionnaire (PCQ-R for Chinese) was used [33]. Combined with the characteristics of nursing, Chinese scholar Luo revised the PCQ-R from the Psychological Capital Questionnaire (PCQ) developed by Luthans et al. [13]. The PCQ-R consisted of 20 items in 4 dimensions: self-efficacy (6 items), hope (6 items), resilience (5 items), and optimism (3 items). Each item is rated on a 6-point Likert scale, with higher scores indicating higher levels of psychological capital. The reliability of the instrument in this study is 0.979.

#### Organizational identification

To measure organizational identification, the Chinese version of Organizational Identification Questionnaire developed by Li [34] was used. The questionnaire consists of 8 items, and each item is rated on a 5-point Likert scale, with higher scores indicating higher levels of organizational identification. The reliability of the instrument in this study is 0.954.

### Data analysis

SPSS 22.0 and AMOS 24.0 were used in this present study for data analysis. A two-step strategy was taken to analyze the data collected in this study [35]. First, confirmatory factory analysis (CFA) was used to compare the model fit the hypothesized model and the other three alternative models to explore whether all variables were distinguishable or not. In addition, common method bias was tested to identify the risk of common method bias from self-reported questionnaire survey. Secondly, structural equation models were performed to test whether the association between organizational support and task/contextual performance was mediated by psychological support and organizational identification. In the second procedure of data analysis, the direct effect of organizational support on nurses’ task/contextual performance without inclusion of the mediators, as well as the indirect mediation effects and direct effects of psychological support and organizational identification by bootstrapping method were also examined.

### Ethical considerations

We submitted the research protocol of this study to the Medical Ethics Committee of Army Medical University. A determination of exemption was made by the Medical Ethics Committee.

## Results

### Descriptive statistics and correlations

Mean and standard deviations of organizational support, psychological capital, organizational identification, task/contextual performance were shown in Table 2. The results of the correlation analysis were also shown in Table 2. There was a significant correlation between the above variables.

**Table 2:** Means, standard deviations, and correlations among the study variables

Variables	OS	PC	OI	TP	CP
OS	1				
PC	0.73***	1			
OI	0.72***	0.71***	1		
TP	0.48***	0.66***	0.58***	1	
CP	0.57***	0.66***	0.73***	0.72***	1
Mean	4.07	5.04	4.45	4.20	4.49
standard deviation	0.79	0.72	0.56	0.60	0.48

***P < 0.01

Note: OS—organizational support, PC—psychological capital, OI—organizational identification, TP—task performance, CP—contextual performance

### Confirmatory factor analysis

Confirmatory factor analysis of comparison between the five-factor model, four-factor model, and single-factor model were performed, respectively. The five-factor models used in this study were the most suitable by comparing fitting indices.

### Common method deviation test

Harmon Single Factor Test [36] was also performed under the condition that organizational support, psychological capital, organizational identification, task performance and contextual performance were included as a single latent factor. The result showed that the single factor model (CFI = 0.65, RMSEA=0.27) fit poorly to the data in this study. Additionally, a latent common method factor was included in the confirmatory factor analysis [36], and the fit indices of this model were compared with the indices of the original five-factor model. The difference in CFI and TLI was less than 0.1, indicating that there was no common method bias.

### Structural equation model analysis

Firstly, structural equation model 1 was developed with organizational support being the independent variable and task performance being the dependent variable, and structural equation model 2 was developed with organizational support being the independent variable and contextual performance being the dependent variable at the same time. The direct effect of model 1 and model 2 was examined, and the model fitting of model 1 and model 2 (Table 3) was good. Direct effect test found that organizational support could positively affect task performance (β = 0.51, p < 0.001) and contextual performance (β = 0.61, p < 0.001), which supported Hypothesis 1.1 and Hypothesis 1.2.

Secondly, the structural equation model 3 and model 4 were developed, with organizational support being the independent variable, task performance being the dependent variable, and psychological capital and organizational identification being the single mediators, respectively. In the meanwhile, the structural equation model 5 and model 6 were developed with organizational support being the independent variable, contextual performance being the dependent variable, and psychological capital and organizational identification being the single mediators, respectively. The model fitting of model 3, model 4, model 5, and model 6 (Table 3) was good.

The mediation effects were tested using the bootstrap method with 1000 bootstrap resamples. The mediation effect of psychological capital on task performance and contextual performance was 0.54 (95% CI: 0.44, 0.65) and 0.45 (95% CI: 0.36, 0.56), which supported Hypothesis 2.1 and Hypothesis 2.2, respectively. The mediation effect of organizational identification on task performance and contextual performance was 0.41 (95% CI: 0.27, 0.53) and 0.37 (95% CI: 0.30, 0.44), which supported Hypothesis 3.1 and Hypothesis 3.2, respectively. The mediation effect of contextual performance on organizational support and task performance was 0.49 (95% CI: 0.40, 0.59) .

**Fig. 2:**
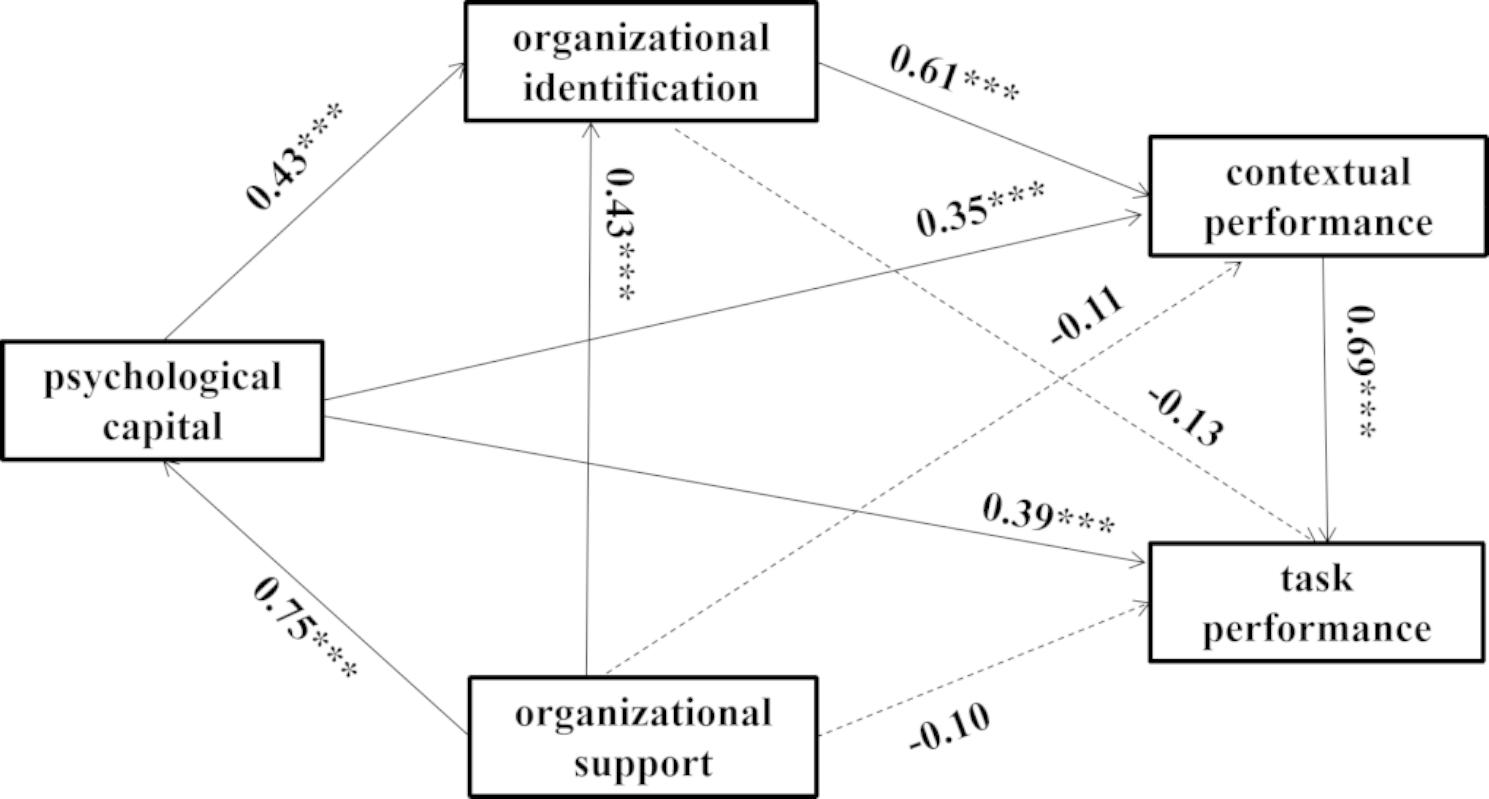
Path results of hypothesized model, ***P < 0.01

Third, the chain mediation effect of psychological capital and organizational identification on the relationship between organizational support on task/contextual performance was examined by Hayes’ multiple mediation method [37], as well as the chain mediation effect of psychological capital, organizational identification, and contextual performance on the relationship between organizational support and task performance. This was also achieved by the bootstrap method with 1000 bootstrap resamples, and the results can be seen in Table 4; Fig. 2. The multi-mediating effect of the path (organizational support→psychological capital→organizational identification→task performance) was 0.14 (95% CI: 0.01, 0.25), which supported Hypothesis 4.1. The multi-mediating effect of the path (organizational support→psychological capital→organizational identification→contextual performance) was 0.25 (95% CI: 0.18, 0.32), which supported Hypothesis 4.2. The multi-mediating effect of the path (organizational support→psychological capital→organizational identification→contextual performance→task performance) was 0.21 (95% CI: 0.09, 0.32), which supported Hypothesis 4.3.

**Table 3:** Results of the multiple mediation model

Path	Model 1	Model 2	Model 3	Model 4	Model 5	Model 6	Hypothesized model
OS→TP	0.51***						−0.10
OS→CP		0.61***					−0.11
OS→PC→TP			0.54***				0.21***
OS→OI→CP					0.41***		−0.04
OS→PC→TP				0.45***			0.17***
OS→OI→CP						0.37***	−0.04
OS→PC→OI→TP							0.14***
OS→PC→OI→CP							0.25***
OS→PC→OI→CP→TP							0.21***
Model Fit Indices	χ2/df =2.43 CFI = 1.00 TLI = 1.00 RMSEA = 0.06	χ2/df = 1.45 CFI = 1.00 TLI = 1.00 RMSEA = 0.03	χ2/df = 3.88 CFI = 0.99 TLI = 0.98 RMSEA = 0.08	χ2/df = 4.28 CFI = 0.98 TLI = 0.98 RMSEA = 0.09	χ2/df = 3.67 CFI = 0.99 TLI = 0.98 RMSEA = 0.08	χ2/df = 2.98 CFI = 0.99 TLI = 0.99 RMSEA = 0.07	χ2/df = 3.40 CFI = 0.98 IFI = 0.97 RMSEA = 0.07

Note: Regression coefficients are standard values; OS—organizational support, PC—psychological capital, OI—organizational identification, TP—task performance, CP—contextual performance

***P < 0.01

**Table 4:** Results of the multiple mediation model

Effect	path	Effect value	Boot standard error	95% confidence interval	
				Upper limit	Lower limit
Direct effect	OS→TP	−0.10	0.07	−0.23	0.03
	OS→CP	−0.11	0.07	−0.25	0.01
Indirect effect	OS→PC→TP	0.21	0.03	0.14	0.29
	OS→OI→TP	−0.04	0.04	−0.11	0.01
	OS→PC→CP	0.17	0.04	0.10	0.24
	OS→OI→CP	−0.04	0.03	−0.10	0.00
	OS→PC→OI→TP	0.14	0.06	0.02	0.25
	OS→PC→OI→CP	0.25	0.03	0.18	0.32
	OS→PC→OI→CP→TP	0.21	0.06	0.09	0.32

Note: OS—organizational support, PC—psychological capital, OI—organizational identification, TP—task performance, CP—contextual performance

## Discussion

This study aimed to evaluate the chain mediating effect of psychological capital and organizational identification on the relationship between organizational support and job performance of nurses in public hospitals in China. In this study, associations between organizational support, psychological capital, organizational identification, task/contextual performance were analyzed. The mechanism of how psychological capital and organizational identification mediated the link between organizational support and task/contextual performance was also examined.

First, this study supports past findings that there was a positive correlation between organizational support and job performance among nurses [24]. Organizational support can not only contribute to physical and emotional attachment of employees to the organization [8, 9], but can also promote the balance between work and life [38]. According to organization support theory, when employees perceive more support from their organizations, they would reveal higher commitment to their organizations and achieve better performance [29, 39]. According to the social change theory, when an organization can provide more benefits to employees, they also can react with better performance [40]. Job performance is one of the main outcomes of organizational support [39], which is also found in many studies of different populations [41]. Nurses face tremendous psychological pressure to ensure patient safety and quality nursing care [42], and their job performance is a priority issue in healthcare organizations [43]. Nurse managers therefore focus on creating a positive work environment for nurses and value the development of strong organizational support. From this study, it can be seen that organizational support is not only significantly related to nurses’ task performance, which is consistent with past research [10], but also is significantly related to nurses’ contextual performance. In the meanwhile, task performance and contextual performance were positively correlated in this study, which was also consistent with theory of job performance [3].

After examining the mediation effects of psychological capital and organizational identification as single mediators between organizational support and task/contextual performance, it can be found that the mediating role of psychological capital was well grounded. This research indicates that the higher organizational support, the higher the psychological capital, which generates a higher level of task/contextual performance among nurses, which is consistent with past research [41]. In addition, Chinese female nurses who have more organizational support are reported to have a high level of psychological capital, which can also generate more work engagement [44]. As nursing has been identified as a common high-risk profession, the value of psychological capital as personal strength gains wide acknowledgement in the field of nursing management [45]. The main participants in the study are female, and the importance of psychological capital in generating better job performance is demonstrated in this sample. The concern is that meta-analysis shows that nurses in Asia are reported to have the lowest level of psychological capital [46]. In the meanwhile, the level of psychological capital are at risk of declining from 2019 to 2021 [46]. Therefore, policymakers and nurse managers should initiate more psychological training programs or formulate better strategies to sustain psychological capital among nursing personnel.

In addition, this study demonstrated the mediating role of organizational identification. It is shown that the higher the organizational support, the higher the organizational identification, which could generate a higher level of task/contextual performance among nurses. The above finding was consistent with past research among company employees [22]. Notably, this is the first study to demonstrate the mediation effect of organizational identification on the relationship between organizational support and job performance. Organizational identification as a predictor of turnover should be valued and cultivated, especially among new graduate nurses [47]. Moreover, nurse managers play a crucial role in creating a stable organizational identity, especially head nurses are reported to have a strong influence on organizational culture in the ward [48]. Therefore, it is critical that nurse managers and health organizations understand the importance of organizational vision.

In this study, the results of the chain mediation model show that psychological capital and organizational identification play a mediating role in the relationship between organizational support and nurses’ job performance, respectively. In addition, psychological capital, organizational identification, and contextual performance play a mediating role in the relationship between organizational support and nurses’ task performance. However, the results in Fig. 2 also indicate that organizational support could not directly affect nurses’ task performance when psychological capital, organizational identification, and contextual performance are included in the analysis. These findings highlight the crucial role of psychological capital and organizational identification in improving task performance and contextual performance. The above findings of the chain mediation model are interesting, which can lead us to develop strategies for job performance from an organizational perspective.

Nurses make up the largest workforce in healthcare organizations, and job performance of nurses determines the overall performance of healthcare organizations [43]. For nurse managers, providing adequate organizational support, improving psychological capital, and enhancing organizational identification is important for improving nurses’ job performance. First, nursing managers should provide an organizational culture of supportive work environments for nurses, which promotes psychological capital, fosters organizational identification, minimizes stressors, and reduces workload. Secondly, nursing managers can design more training programs such as the Psychological Capital Intervention Model [49] to promote psychological capital to face the challenges of clinical nursing. Third, nursing managers should pay attention to organizational identification to increase nurses’ awareness of belonging, especially among new graduate nurses.

This research has several limitations that can be considered in the future. Firstly, a cross-sectional study was carried out to provide a theoretical basis for increasing the job performance of nurses. Future studies should take the longitudinal data into account which could be used to examine the mechanism of organizational support’s effect on job performance among nurses. Secondly, since this study was conducted through self-reported surveys, it is necessary to explore other sources of research data to enrich the scope of the model built in this study. Third, the setting of this study was public hospital. Considering organizational support in other settings such as private clinics or community health centers would be different, and this area needs to to be explored in future research. Finally, it may be worthwhile to explore the generalization of the findings of this study to other populations and countries. Future research should therefore verify the generalization of our findings with empirical data collected from different regions and countries.

## Conclusion

This study provided evidence of how organizational support determined nurses’ job performance by providing a chain moderating role of psychological capital and organizational identification. The findings revealed that there was a significant positive relationship between the above variables in this study, which provided guidance for developing strategies to improve job performance among nurses and other personnel in healthcare organizations. Nursing managers should pay attention to the change of nurses’ psychological capital and organizational identification, and make efforts to enhance their organizational support at the same time. In addition, nursing managers can make efforts to provide a supportive work environment in healthcare organizations by enhancing organizational support, psychological capital, and organizational identification as a whole. In the meanwhile, limitations of this study, such as cross-sectional design and self-report measures, should be addressed. In the future, longitudinal studies and interventions could be carried out in this area so as to provide more empirical evidence for hospital management.

## Electronic supplementary material

Below is the link to the electronic supplementary material.

Supplementary Material 1

## Data Availability

The datasets used and/or analyzed during the current study are available from the corresponding author on reasonable request.

## Abbreviations

CFA: Confirmatory factory analysis
JD-R-S: Demands-resources-stressors
PCQ: Psychological Capital Questionnaire.
