# Supplementary material for: Assessing the effects of organizational support, psychological capital, organizational identification on job performance among nurses: a structural equation modeling approach
Source: BMC Health Serv Res. 2023 Jul 27;23:806. doi: 10.1186/s12913-023-09705-z (PMC10375763; doi:10.1186/s12913-023-09705-z)
Supplement: Supplementary file 1 — Supplementary Material 1 [file 12913_2023_9705_MOESM1_ESM.docx]

| **Appendix. Scale items** | |
| --- | --- |
| Perceived organizational support | |
|  | The hospital values my contribution to its well-being. |
|  | The hospital really cares about my well-being. |
|  | The hospital can listen to my opinions. |
|  | When I encounter difficulties in my work, the hospital try its best to help me. |
|  | When I encounter difficulties in my life, the hospital try its best to help me. |
|  | The hospital provide me with opportunities for promotion. |
|  | The hospital will respect my goals and values. |
|  | The hospital cares about my personal development. |
|  | The hospital cares about my health. |
|  | The hospital try its best to solve the worries of my life and family. |
|  | The hospital try its best to provide me with a good working environment. |
|  | The hospital try its best to provide me with the tools and information needed for my work. |
|  | The hospital try its best to provide me with relevant training for my work. |
| Task performance | |
|  | I have made significant contributions to the work of our department (or unit). |
|  | I can always finish the tasks assigned to me on time. |
|  | I am one of the best employees in our department (or unit). |
|  | My work achievements always meet the expectations of my superiors. |
| Contextual performance | |
|  | I would praise colleagues when they succeed. |
|  | When colleagues encounter personal difficulties, give them support or encouragement. |
|  | When a certain practice may affect colleagues, I will tell them in advance. |
|  | I only talk about things that are beneficial to colleagues or groups. |
|  | I encourage others to overcome interpersonal barriers and get along well. |
|  | I always treat others fairly. |
|  | I always take the initiative to help others. |
|  | I use my break time to work to ensure that the task is completed on time. |
|  | I pay attention to the important details of my work. |
|  | I work extra hard. |
|  | I look for a challenging job. |
|  | I have self-discipline at work. |
|  | I take the initiative to solve problems at work. |
|  | I am determined to overcome difficulties and complete my tasks. |
|  | I actively and enthusiastically solve difficult work. |
| Psychological capital | |
|  | I believe I can analyze long-term problems and find solutions. |
|  | At the meeting, I am confident in stating things within my scope of work. |
|  | I believe that I have contributed to the discussion on the development of hospital nursing. |
|  | Within my scope of work, I believe I can help set goals. |
|  | I believe I can contact and discuss problems with people outside the department. |
|  | I believe I can present information to colleagues. |
|  | In difficult situations I think I am able to find the way out. |
|  | I am full of energy to complete my work objectives. |
|  | There are many solutions to any problem. |
|  | I think I am quite successful in my work. |
|  | I can think of many ways to achieve my current work goals. |
|  | I am achieving the work goal I set for myself. |
|  | In my work, I will solve the problems I encounter anyway. |
|  | If I have to do it at work, I can also fight independently. |
|  | I usually take the pressure at work calmly. |
|  | Because I have experienced many hardships before, I can survive the difficult period in my work now. |
|  | In my current work, I feel that I can handle many things at the same time. |
|  | I always see the bright side of things in my work. |
|  | I am optimistic about what will happen to my work in the future. |
|  | I always try to believe that behind every cloud there is the blue sky. |
| Organizational identification | |
|  | I think my values are consistent with those of the hospital. |
|  | On the whole, I agree with the organization. |
|  | I am proud of being a member of the hospital. |
|  | I am very happy to spend my career in my current organization. |
|  | I am very concerned about others' evaluation of the unit. |
|  | I think the unit is like a warm big family. |
|  | I work hard for the unit. |
|  | I’m glad to hear that others envy my work. |
